# Structural and Magnetic Property of Cr^3+^ Substituted Cobalt Ferrite Nanomaterials Prepared by the Sol-Gel Method

**DOI:** 10.3390/ma11112095

**Published:** 2018-10-25

**Authors:** Jinpei Lin, Jiaqi Zhang, Hao Sun, Qing Lin, Zeping Guo, Hu Yang, Yun He

**Affiliations:** 1 Guangxi Key Laboratory of Nuclear Physics and Nuclear Technology, Guangxi Normal University, Guilin 541004, China; linjinpei.2007@163.com (J.L.); linqinglab@126.com (J.Z.); heyunlab@163.com (H.S.); 2 College of Medical Informatics, Hainan Medical University, Haikou 571199, China; 3 College of Physics and Technology, Guangxi Normal University, Guilin 541004, China; zepingguo@mailbox.gxnu.edu.cn (Z.G.); yanghu000@126.com (H.Y.); 4 Sate Key Laboratory for Chemistry and Molecular Engineering of Medicinal Resources, Guangxi Normal University, Guilin 541004, China

**Keywords:** Co-Cr-Ferrite, sol-gel auto-combustion, nanoparticle structure, Mössbauer, superparamagnetic

## Abstract

Cobalt-chromium ferrite, CoCr*_x_*Fe_2−*x*_O_4_ (*x =* 0*–*1.2), has been synthesized by the sol-gel auto-combustion method. X-ray diffraction (XRD) indicates that samples calcined at 800 °C for 3 h were a single-cubic phase. The lattice parameter decreased with increasing Cr concentration. Scanning electron microscopy (SEM) confirmed that the sample powders were nanoparticles. It was confirmed from the room temperature Mössbauer spectra that transition from the ferrimagnetic state to the superparamagnetic state occurred with the doping of chromium. Both the saturation magnetization and the coercivity decreased with the chromium doping. With a higher annealing temperature, the saturation magnetization increased and the coercivity increased initially and then decreased for CoCr_0.2_Fe_1.8_O_4_.

## 1. Introduction

Ferrite is an important magnetic material. Cobalt ferrite is a hard magnetic ferrite. It has a high coercivity of 5000 Oe, a moderate saturation magnetization of about 80 emu/g, a high Curie temperature (T_C_) of 520 °C, and a high anisotropy constant of 2.65 × 10^6^ to 5.1 × 10^6^ erg/cm^3^ [1,2]. Moreover, cobalt ferrite exhibits good insulation, and excellent chemical stability and physical hardness [1,2,3]. Hard magnetic cobalt ferrite has been widely used as a high-density magnetic recording medium in magnetic recording [4].

It is well known that the chromium ions can control the magnetic parameters of developing technological materials. Cr^3+^ ions that are substituted for Fe^3+^ ions will change magnetic properties markedly due to their nonmagnetic nature [5,6]. Kwang et al. [6] studied the magnetic properties of ferrite powders, such as CoCr*_x_*Fe_2−*x*_O_4_, with increasing Cr content, and the Mössbauer spectra varied from Zeeman sextets to a relaxed doublet for CoCr*_x_*Fe_2−*x*_O_4_. The Mössbauer spectra of non-magnetic Zn^2+^ ions substituted for Co-ferrite also transitioned from sextets to doublets [7,8]. Muhammad et al. [9] prepared CoCr*_x_*Fe_2−*x*_O_4_ nanomaterials using the micro-emulsion method, and the Direct current (DC)-electrical resistivity decreased as the temperature increased, indicating the semiconductive nature of the ferrites. Yüksel et al. [10] synthesized nanocrystalline chromium substituted cobalt ferrite powders by a hydrothermal route. Man et al. [11] investigated the magnetic properties and quantum couplings of spinel ferrite CoCr*_x_*Fe_2−*x*_O_4_ nanoparticles. However, ferrite powders synthesized via the sol-gel auto-combustion method have good sinterability due to their homogeneous composition and have other advantages of using simple equipment and low material cost [5]. By choosing an appropriate complexing agent and chemical additives, as well as a suitable atmosphere and heating source, it has been possible to affect crystallinity, phase purity, particle size, and defect concentration, thereby influencing the magnetic properties of the ferrite [12].

In the present study, ferrite, CoCr*_x_*Fe_2−*x*_O_4_ (*x =* 0*–*1.2), powders were prepared using the sol-gel auto-combustion process. We have investigated the influence of doping content and calcining temperature on the structure by X-ray diffraction and scanning electron microscopy, and studied the magnetic properties of the different calcining temperatures and doping content samples by Mössbauer spectroscopy and a Quantum Design MPMS-XL7 magnetometer. The research purpose is to provide a solid base of knowledge for the synthesis of cobalt ferrite powders that have excellent magnetic properties.

## 2. Methodology

### 2.1. Preparation of Samples

Chromium substituted cobalt ferrite, CoCr*_x_*Fe_2−*x*_O_4_ (*x =* 0*–*1.2), powders were prepared by a sol-gel auto-combustion method. Analytical grade chemicals, including cobalt nitrate, Co(NO_3_)_2_·6H_2_O; chromium nitrate, Cr(NO_3_)_3_·9H_2_O; iron nitrate, Fe(NO_3_)_3_·9H_2_O; citric acid, (C_6_H_8_O_7_·H_2_O); and ammonia, (NH_3_·H_2_O), were used as raw materials, and the citric acid was used as a complexing agent for the spinel ferrite synthesis. The molar ratio of metal nitrates to citric acid was 1:1. Nitrate and citric acid were dissolved in deionized water, respectively. The pH of the metal nitrate was adjusted to an appropriate pH of 7 to 9 by the addition of aqueous ammonia. The mixed solution was placed in an 80-degree water bath for heating in a water bath, and citric acid was gradually added dropwise during the water bath, and the mixed solution was continuously stirred until a wet gel was formed. The wet gel was dried in a digital drying oven at 120 °C for 2 h, and the obtained dry gel was ignited in the air with a combustion improver (anhydrous ethanol) to ignite the self-propagating powder to obtain a fluffy powder. After the powder was ground, the final sample was obtained by calcination at two specific temperatures (400 °C and 800 °C) for 3 h in a muffle furnace.

### 2.2. Characterization

The crystal structure of the sample was analyzed by an X-ray diffractometer (D/max-2500 V/PC, Rigaku, Japan) using a Cu Kα target (λ = 0.15405 nm). The morphology of the sample was observed by a scanning electron microscope (NoVaTM Nano SEM 430, Thermo Fisher Scientific, Waltham, MA, USA). The Mössbauer spectrum at room temperature was measured using an isothermal accelerated Mössbauer spectrometer (Fast Com Tec PC-mossⅡ, Oberhaching, Germany) with a source of ^57^Co and labeled with α-Fe. Measurement of hysteresis loops of samples at room temperature was completed using a superconducting quantum interference magnetometer (MPMS-XL-7, Quantum Design, San Diego, CA, USA). 

## 3. Results and Discussion

### 3.1. X-ray Diffraction Analysis

**Figure 1:**
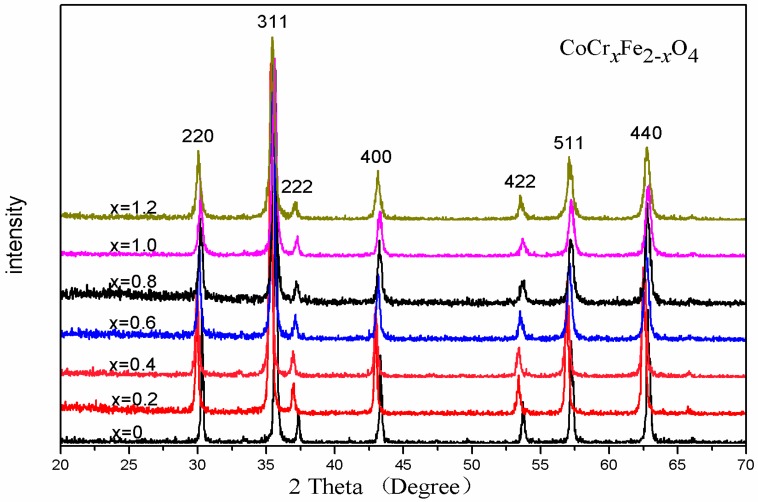
XRD patterns of CoCr*_x_*Fe_2-*x*_O_4_ calcined at 800 °C.

Figure 1 shows the XRD diffraction pattern of CoCr*_x_*Fe_2−*x*_O_4_ (*x =* 0*–*1.2) ferrite after calcination at 800 °C for 3 h. The results show that all of the ferrites had a single-phase structure. Other impurity peaks were not detected for these samples. Thus, it can be seen that Cr^3+^ ion substituted the Fe^3+^ ion at the octahedral (B) site and was not influenced by the spinel cubic structure of the cobalt ferrite. When 0 < x < 1, the trend of the lattice parameters was to decrease with increasing chromium concentration, as shown in Table 1. The decrease in the lattice parameter can be attributed to the substitution of the larger Fe^3+^ ions (0.645 Å) by smaller Cr^3+^ ions (0.63 Å) [9]. When 1 < x, the increase of lattice parameter is attributed to the elastic strain and magneto-volume effects [12].

The density of X rays is determined by the following formula [13,14,15]:

(1) $$\rho_{x}=\frac{8M}{Na^{3}}$$

where *a* is the lattice parameter, *M* is relative molecular mass, and *N* is the Avogadro number. 

The X-ray density decreased with Cr^3+^ concentration for all samples, as is evident from Table 1. The atomic weight of Fe is greater than that of Cr, so the relative molecular mass decreases with an increasing content of Cr^3+^ ions. The decrease in X-ray density was attributed to the fact that the relative molecular mass decreased more than the negligible decline of the lattice constant. 

**Figure 2:**
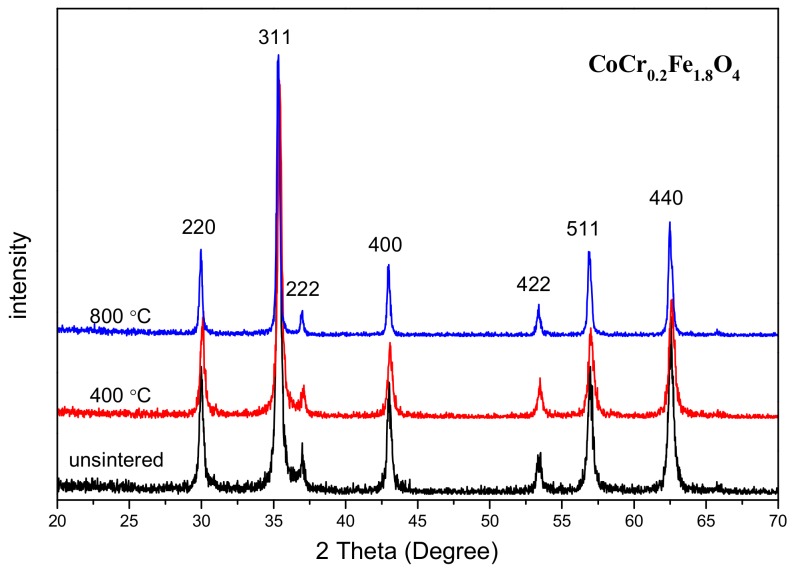
XRD patterns of CoCr_0.2_Fe_1.8_O_4_ calcined at different temperatures.

The average crystallite size consistently decreased with increasing Cr content, as is evident from Table 1, and similar results have been reported in the literature [6]. Figure 2 shows the X-ray diffraction pattern of the sample CoCr_0.2_Fe_1.8_O_4_ when calcined at different temperatures. All samples were single-phase spinel structures with no heterophases found. The lattice constants of all samples did not show much change. The average crystallite size of CoCr_0.2_Fe_1.8_O_4_ increased with increasing calcining temperature as can be observed from Table 2. 

In other published work [14], the diffraction peaks of Ni_0.50_Cu_0.25_Zn_0.25_Cr*_x_*Fe_2−*x*_O_4_ calcined at lower temperatures were not very sharp, but in the present study, the diffraction peaks of unsintered CoCr_0.2_Fe_1.8_O_4_ were very sharp indeed. The chromium-replaced cobalt ferrite powders were prepared using the sol-gel auto-combustion process, and before calcining, the samples had excellent crystallinity. 

### 3.2. Scanning Electron Microscopy (SEM) 

**Figure 3:**
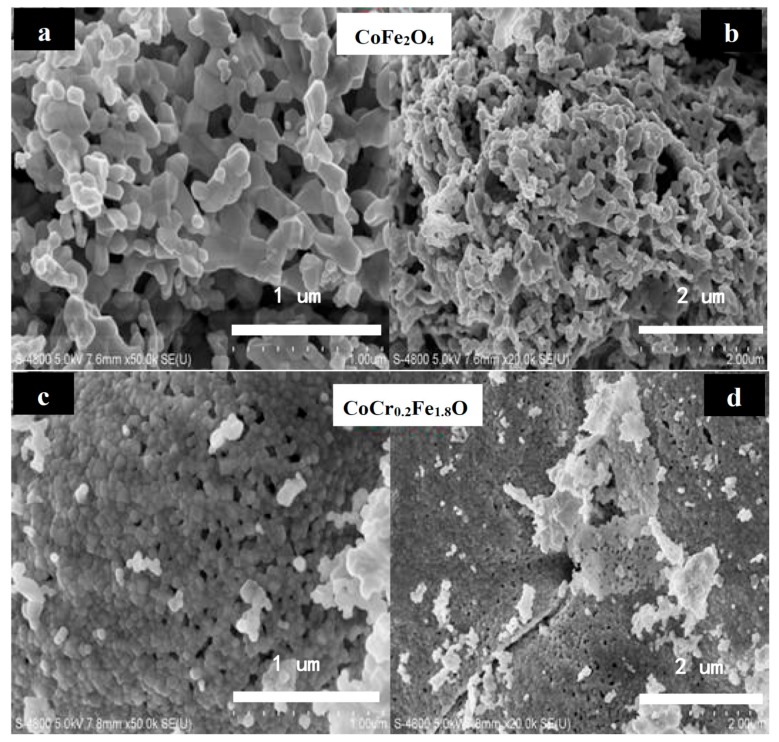
SEM micrographs depict of CoFe_2_O_4_ (*x =* 0) and CoCr_0.2_Fe_1.8_O_4_ (*x =* 0.2) ferrites with diameters of 1 um (**a**), 2 um (**b**), 1 um (**c**), and 2 um (**d**).

**Figure 4:**
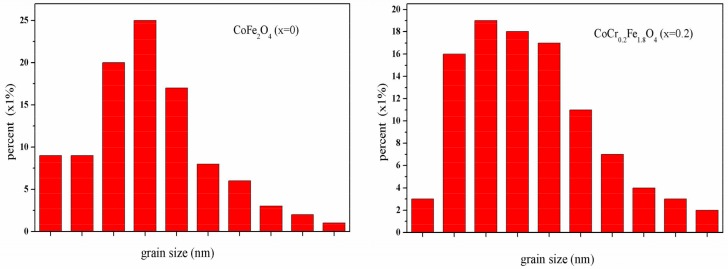
The grain size distributions of CoFe_2_O_4_ and CoCr_0.2_Fe_1.8_O_4_ after calcination at 800 °C.

The SEM micrographs of CoCr*_x_*Fe_2−*x*_O_4_ (*x =* 0, 0.2) ferrites annealed at 800 °C for 3 h are shown in Figure 3. It can be observed that the grain size is almost uniform and the material is well crystallized for CoCr*_x_*Fe_2−*x*_O_4_ (*x =* 0, 0.2). Histograms of the comparative grain size distributions of CoFe_2_O_4_ (*x =* 0) and CoCr*_x_*Fe_2−*x*_O_4_ (*x =* 0, 0.2) ferrites are shown in Figure 4. The average particle sizes of CoFe_2_O_4_ and CoCr*_x_*Fe_2−*x*_O_4_ were estimated by a statistical average method to be approximately 96.26 and 51.76 nm, respectively. This shows that the prepared ferrite powders were nanoparticles and that the average grain size decreased significantly by increasing the Cr content, which can be due to the ionic radius of iron atoms being larger than the ionic radius of chromium atoms. The average crystallite size was determined by XRD to be slightly smaller than the average grain size [15].

### 3.3. Mössbauer Spectroscopy

**Figure 5:**
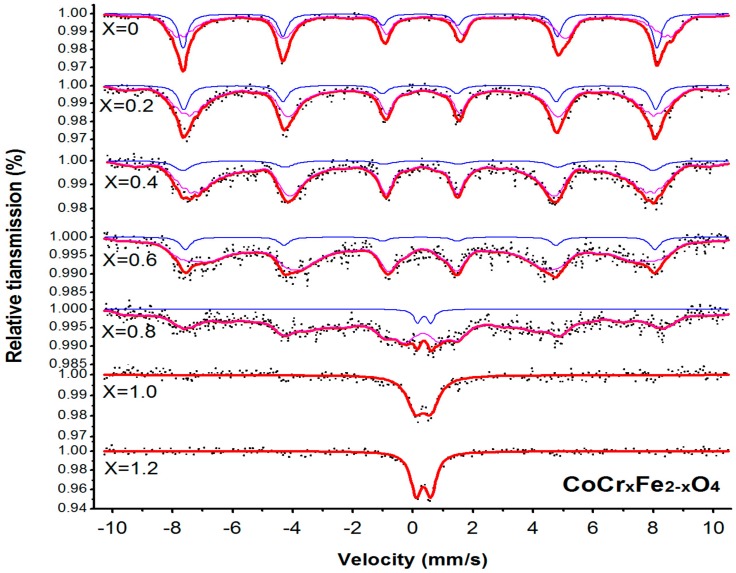
Room temperature Mössbauer spectra for CoCr*_x_*Fe_2-*x*_O_4_ calcined at 800 °C.

The room temperature Mössbauer spectra for CoCr*_x_*Fe_2−*x*_O_4_ are shown in Figure 5. The data of all samples were analyzed using Mösswinn 3.0 software. When 0 ≤ x ≤ 0.6, the spectrum of the sample CoCr*_x_*Fe_2−*x*_O_4_ is fitted with two sets of six-line peaks, because Fe^3+^ takes the tetrahedral A at the B-position of the octahedron. This indicates the ferromagnetic properties of the sample. Among them, the six-line peak of the B position is larger in the isomer shift (I.S.), and the six-line peak of the A position is smaller. This is because the bond length of the tetrahedral A site Fe^3+^_O^2−^ is smaller than the bond length of the octahedral B site Fe^3+^_O^2−^, and the orbital overlap of the A site Fe^3+^_O^2−^ is larger; that is, the A site is more covalent than the B site. The covalentity is large, so the isomer shift (I.S.) of the B position is larger [6]. Other studies have shown that the isomer shift (I.S.) value of Fe^3+^ ions is 0.1 to 0.5 mm/s, while for Fe^2+^, it is 0.6 to 1.7 mm/s [16]. From Table 3, values for I.S. in the present study indicate that iron is in the Fe^3+^ state.

The values of the magnetic hyperfine field at the A and B sites decrease with increasing chromium substitution, and for the hyperfine field of the octahedral B site, the decrease is more evident, as shown in Table 3. The observed decrease in the octahedral magnetic hyperfine field with the increasing of Cr^3+^ ions is due to the decrease in the A-B exchange interactions, which results in the lower magnetic hyperfine field [6,17]. In all the samples, the quadrupole shift of the A and B magnetic sextet was very small, which indicated that the ferrites were in close cubic symmetry [18,19].

For the CoCr*_x_*Fe_2−*x*_O_4_ with *x =* 0.8, the Mössbauer spectrum exhibits the characteristics of the Magnetic effect, which consists of a set of six-line peaks and a set of paramagnetic doublets. The samples transformed into a mixed state of superparamagnetic and magnetic orders from the initial magnetic order. Mössbauer spectra of the samples with *x =* 1.0, 1.2 consisted only of a central doublet, and it exhibited super-paramagnetic characteristics. This was attributed to the reduction of the magnetic interactions between iron ions with greater Cr^3+^ dilution. The decrease in the line width of this doublet exhibited increased relative intensity due to the increase in the substituted Cr content [17].

**Figure 6:**
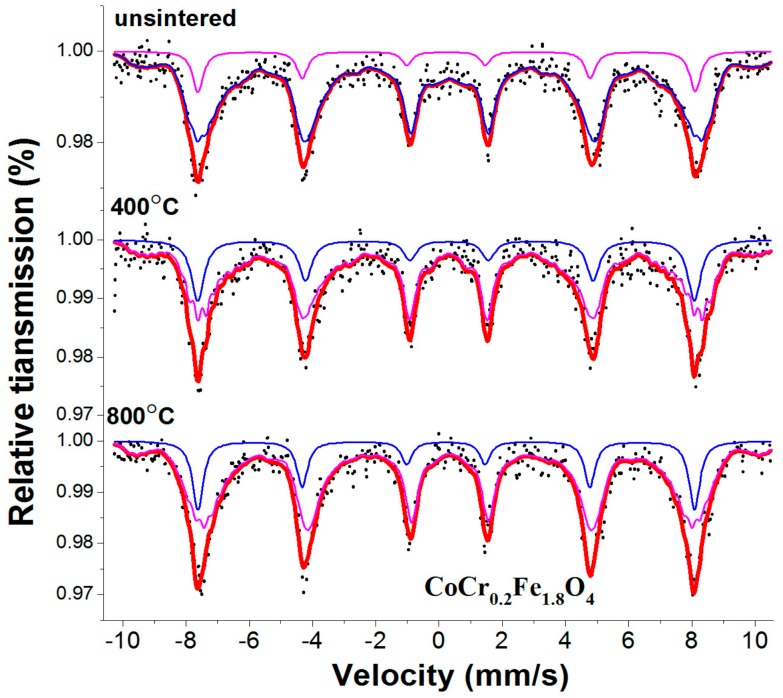
Room temperature Mössbauer spectra for CoCr_0.2_Fe_1.8_O_4_ calcined at different temperatures.

The room temperature Mössbauer spectrum of CoCr_0.2_Fe_1.8_O_4_ calcined at different temperatures are displayed in Figure 6. All the Mössbauer spectra were fitted with two sextet sub-patterns. Table 4 shows the magnetic hyperfine field increased slightly with the annealing temperature. The results of XRD analysis showed that the samples were well crystallized at different temperatures, and the average grain size became larger as the calcination temperature increased. The magnetic hyperfine field of the sample increases as the calcination temperature increases. Therefore, the change of the magnetic hyperfine field can be explained as the average grain size caused by the change of the calcination temperature [8]. The absorption area of Mössbauer energy for CoCr_0.2_Fe_1.8_O_4_ calcined at different temperatures exhibited certain changes, which indicated that the calcining temperature influenced the fraction of the Fe^3+^ ions in the tetrahedral A and octahedral B sites [6,20,21].

### 3.4. Magnetic Analysis

**Figure 7:**
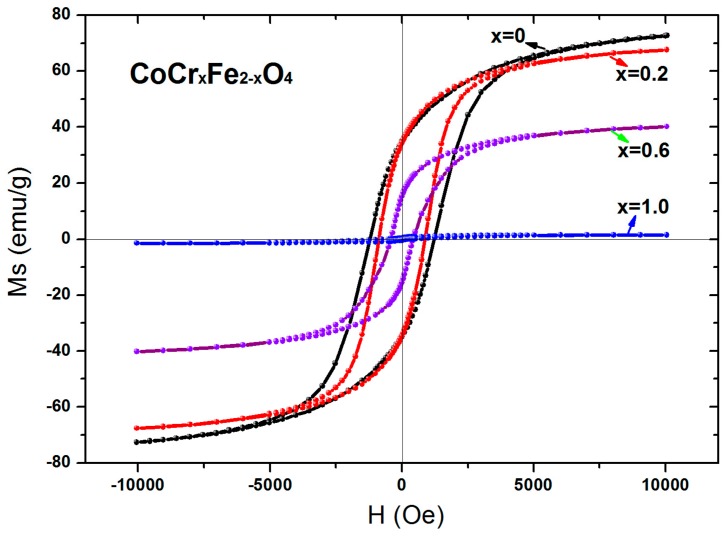
Room temperature magnetic hysteresis loops for CoCr*_x_*Fe_2−*x*_O_4_ calcined at 800 °C.

The magnetic hysteresis loops for CoCr*_x_*Fe_2−*x*_O_4_ at room temperature are shown in Figure 7. For all samples, magnetization reached saturation at a magnetic field of 10,000 Oe. It can be observed from Table 5 that the saturation magnetization of the sample decreases with the substitution of chromium ions. The saturation magnetization of the sample is expressed as the following relationship [5]:

(2) $$\sigma_{s}=\frac{5585\times n_{B}}{M}$$

where *n_B_* is the magnetic moment in terms of Bohr magnetons, and *M* is the relative molecular mass. With the substitution of chromium ions, the relative molecular mass of the sample CoCr*_x_*Fe_2−*x*_O_4_ decreases, and the change in magnetic moment can be explained by the Nell theory. The ionic magnetic moments of the Cr^3+^, Co^2+^, and Fe^3+^ ions are 3*μ_B_*, 3*μ_B_*, and 5*μ_B_* [5,9,13], respectively. According to the two-sublattice model of Néel’s theory [14,19], using the cation distribution of (Fe)_A_[CoCr*_x_*Fe_1−*x*_]_B_O_4_, the Co^2+^ ions prefer to occupy the octahedral site (B) in the CoFe_2_O_4_ material with an inverse spinel structure [1,2], and Cr^3+^ ions have a tendency to occupy the B-site [5,10,20]. The magnetic moment, n_B_, therefore, can be expressed as [5,10,13]:

*n_B_*= *M_B_*− *M_A_*= 3 + 3*x* + 5(1 − *x*) – 5 = 3 − 2*x* (3)

**Figure 8:**
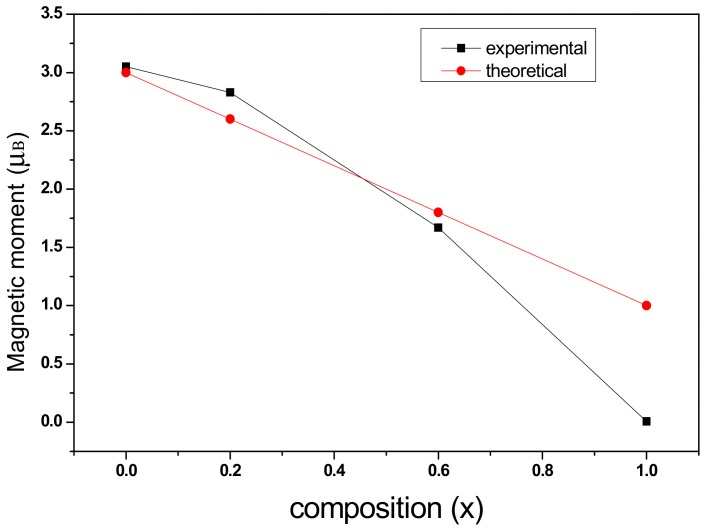
Variation in experimental and theoretical magnetic moments with chromium content (x*).*

The *M_B_* and *M_A_* in the formula are the B-position of the octahedral lattice and the A-site of the tetrahedral lattice [21,22]. Figure 8 shows the variation of the total magnetic moment obtained by theoretical calculations with chromium substitution. 

As can be seen from Figure 8, the experimental magnetic moment and the theoretical magnetic moment decreases as the Cr content, *x*, increases, and according to the relationship (2), the theoretical saturation magnetization decreases with increasing chromium concentration. The saturation magnetization obtained by the experiment is in accordance with the theoretical analysis for all samples.

It can be observed from Table 5 that the coercive force of the sample CoCr*_x_*Fe_2−*x*_O_4_ decreases with the doping of chromium. The large coercivity originates from the anisotropy of the Co^3+^ ions in the octahedral (B) site as a result of their octahedral lattice having strong spin-orbit coupling [9,23]. The decreasing of coercivity associated with the doping of chromium in the CoCr*_x_*Fe_2−*x*_O_4_ ferrite may be attributed to Co^2+^ ion migration to the tetrahedral site, leading to the observed loss in anisotropy. The magnetocrystalline anisotropy of the Cr ferrites was negative [24,25]. Therefore, the magnetic anisotropy decreased with increasing Cr content, which leads to the decrease in coercivity [26,27].

**Figure 9:**
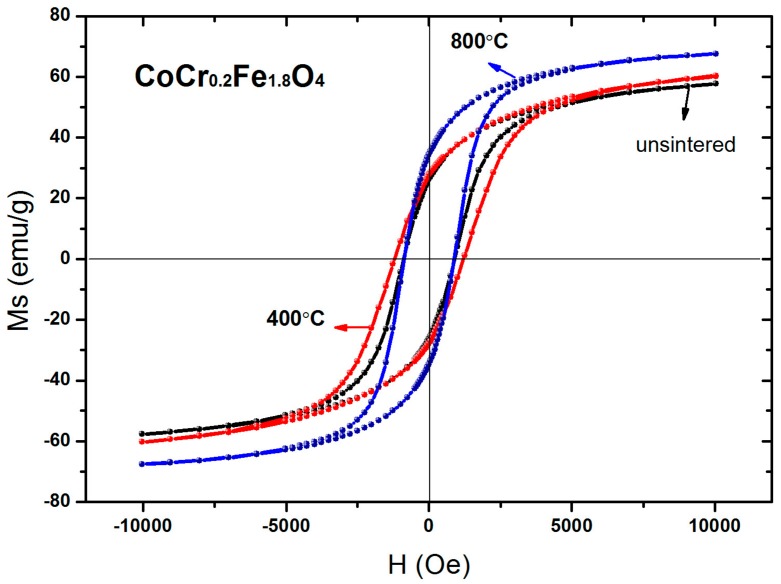
Room temperature hysteresis loops of CoCr_0.2_Fe_1.8_O_4_ calcined at different temperatures.

The room temperature magnetic hysteresis loops for un-sintered CoCr_0.2_Fe_1.8_O_4_ and for the same material after annealing at 400 °C and 800 °C are displayed in Figure 9. The data summarized in Table 6 indicate that saturation magnetization of the CoCr_0.2_Fe_1.8_O_4_ increased with annealing temperature, which was attributed to the increase in particle size [13]. During the annealing process, the net reduction of the free energy of the solid-solid interface and the free energy of the solid-vapor interface is the driving force for grain growth [13,28]. The coercivity of the CoCr_0.2_Fe_1.8_O_4_ initially increased and then decreased as the annealing temperature was increased. This can be explained by the associated variation in grain size [29,30]. In the range of single domain sizes, the coercive force can be expressed as *H_C_*= *g* − *h*/*D^2^*. In the range of multi-domain sizes, the coercive force can be written as *H_C_*= *a* + *b*/*D* as the particle size changes. “*g*, *h*, *a*, *b*” in the formula are constants, and “*D*” is the diameter of the particles [21,31]. Therefore, as the particle size becomes larger in the single domain range, the coercive force becomes larger; and in the multidomain range, as the particle size increases, the coercive force decreases [32,33]. In the present study, the grain size of CoCr_0.2_Fe_1.8_O_4_ calcined at different temperatures may lie in the single domain region or the multi-domain region, so coercivity increases initially and then decreases as the annealing temperature is increased [34].

## 4. Conclusions

The analysis results of the XRD diffraction pattern show that the CoCr*_x_*Fe_2-*x*_O_4_ calcined at 800 °C was a single phase spinel structure. The lattice constant of CoCr*_x_*Fe_2−*x*_O_4_ becomes smaller with the substitution of chromium ions because the radius of chromium ions is smaller than the radius of iron ions. The XRD diffraction pattern of CoCr_0.2_Fe_1.8_O_4_ calcined at different temperatures showed that the sample prepared by the sol-gel self-propagation method had good crystallinity even without calcination. The results of SEM showed that the particles of the prepared sample were distributed in an almost uniform size, and crystallized well. The room temperature Mössbauer analysis of CoCr*_x_*Fe_2−*x*_O_4_ calcined at 800 °C shows that the sample transitions from ferromagnetic to superparamagnetic with the doping of chromium ions. The Mössbauer spectrum of CoCr_0.2_Fe_1.8_O_4_ calcined at different temperatures indicates that the calcination temperature will have an effect on the magnetic properties. Both saturation magnetization and coercivity decreased with the substitution of chromium ions, and the change in saturation magnetization can be explained by the Nell theory. The coercive force becomes smaller with the substitution of chromium ions due to a decrease in the magnetocrystalline anisotropy constant. 

## Figures and Tables

**Table 1:** XRD data for CoCr*_x_*Fe_2-*x*_O_4_ calcined at 800 °C.

Content (*x*)	Lattice Parameter (Å)	Average Crystallite Size(Å)	Density (g/cm^3^)
0	8.35497	556	5.3468
0.2	8.40877	436	5.2250
0.4	8.40790	382	5.2094
0.6	8.38461	340	5.2355
0.8	8.36538	284	5.2543
1.0	8.36368	276	5.2400
1.2	8.37912	244	5.1937

**Table 2:** XRD data for CoCr_0.2_Fe_1.8_O_4_ calcined at different temperatures.

Temperature (°C)	Lattice Parameter (Å)	Average Crystallite Size (Å)	Density (g/cm^3^)
unsintered	8.40551	280	5.2311
400	8.39683	313	5.2473
800	8.40877	436	5.2250

**Table 3:** Mössbauer parameters of isomer shift (IS), quadrupole splitting (QS), magnetic hyperfine field (H), linewidth (Γ), and absorption area (A_0_) for CoCr*_x_*Fe_2−*x*_O_4_ annealed at 800 °C.

Content (*x*)	Component	I.S. (mm/s)	Q.S. (mm/s)	H (T)	Γ (mm/s)	A_0_ (mm/s)
0	Sextet (A)	0.237	−0.004	48.946	0.360	32.4
	Sextet (B)	0.375	−0.024	45.695	0.322	67.6
0.2	Sextet (A)	0.223	0.007	48.724	0.409	20.1
	Sextet (B)	0.317	−0.065	44.454	0.355	79.9
0.4	Sextet (A)	0.222	−0.112	48.581	0.662	11.8
	Sextet (B)	0.303	0.011	43.279	0.347	88.2
0.6	Sextet (A)	0.235	0.010	48.473	0.408	7.6
	Sextet (B)	0.303	−0.059	39.210	0.437	92.4
0.8	Sextet (B)	0.327	0.074	34.844	0.418	97.0
	Double	0.373	0.437	-	0.271	3.0
1.0	Double	0.336	0.520	-	0.663	100
1.2	Double	0.351	0.491	-	0.444	100

**Table 4:** Mössbauer parameters of isomer shift (IS), quadrupole splitting (QS), magnetic hyperfine field (H), linewidth (Γ), and absorption area (A_0_) for CoCr_0.2_Fe_1.8_O_4_ annealed at different temperature.

Temperature (°C)	Component	I.S. (mm/s)	Q.S. (mm/s)	H (T)	Γ (mm/s)	A_0_ (mm/s)
unsintered	Sextet (A)	0.234	0.009	48.790	0.412	11.8
	Sextet (B)	0.336	−0.004	43.424	0.386	88.2
400	Sextet (A)	0.273	−0.104	48.716	0.491	21.8
	Sextet (B)	0.325	0.051	43.557	0.283	78.2
800	Sextet (A)	0.223	0.007	48.724	0.409	20.1
	Sextet (B)	0.317	−0.065	44.454	0.355	79.9

**Table 5:** Magnetic data for CoCr*_x_*Fe_2-*x*_O_4_ calcined at 800 °C.

Content (*x*)	*M_s_*(emu/g)	*H_c_* (Oe)	*M_r_*(emu/g)	*n_B_*
0	72.58	1005.33	34.71	3.05
0.2	67.66	878.10	34.39	2.83
0.6	40.20	401.44	15.51	1.67
1.0	1.51	301.20	0.39	0.006

**Table 6:** Magnetic data for CoCr_0.2_Fe_1.8_O_4_ calcined at different temperatures.

Temperature (°C)	*M_s_*(emu/g)	*H_c_* (Oe)	*M_r_*(emu/g)	*n_B_*
unsintered	57.74	877.06	26.20	2.42
400	60.35	1254.18	27.75	2.53
800	67.66	878.10	34.39	2.83
